# FNDC3B and BPGM Are Involved in Human Papillomavirus-Mediated Carcinogenesis of Cervical Cancer

**DOI:** 10.3389/fonc.2021.783868

**Published:** 2021-12-16

**Authors:** Luhan Zhang, Hong Yu, Tian Deng, Li Ling, Juan Wen, Mingfen Lv, Rongying Ou, Qiaozhi Wang, Yunsheng Xu

**Affiliations:** ^1^ School of Basic Medicine, Southwest Medical University, Luzhou, China; ^2^ Department of Dermatovenerology, The Seventh Affiliated Hospital, Sun Yat-sen University, Shenzhen, China; ^3^ Department of Stomatology, The Seventh Affiliated Hospital, Sun Yat-sen University, Shenzhen, China; ^4^ Department of Dermatovenerology, The First Affiliated Hospital, Wenzhou Medical University, Wenzhou, China; ^5^ Department of Obstetrics and Gynaecology, The First Affiliated Hospital, Wenzhou Medical University, Wenzhou, China

**Keywords:** cervical cancer, WGCNA, FNDC3B, BPGM, human papillomavirus

## Abstract

Human papillomavirus (HPV)-mediated cervical carcinogenesis is a multistep progressing from persistent infection, precancerous lesion to cervical cancer (CCa). Although molecular alterations driven by viral oncoproteins are necessary in cervical carcinogenesis, the key regulators behind the multistep process remain not well understood. It is pivotal to identify the key genes involved in the process for early diagnosis and treatment of this disease. Here we analyzed the mRNA expression profiles in cervical samples including normal, cervical intraepithelial neoplasia (CIN), and CCa. A co-expression network was constructed using weighted gene co-expression network analysis (WGCNA) to reveal the crucial modules in the dynamic process from HPV infection to CCa development. Furthermore, the differentially expressed genes (DEGs) that could distinguish all stages of progression of CCa were screened. The key genes involved in HPV-CCa were identified. It was found that the genes involved in DNA replication/repair and cell cycle were upregulated in CIN compared with normal control, and sustained in CCa, accompanied by substantial metabolic shifts. We found that upregulated fibronectin type III domain-containing 3B (FNDC3B) and downregulated bisphosphoglycerate mutase (BPGM) could differentiate all stages of CCa progression. In patients with CCa, a higher expression of FNDC3B or lower expression of BPGM was closely correlated with a shorter overall survival (OS) and disease-free survival (DFS). A receiver operating characteristic (ROC) analysis of CIN and CCa showed that FNDC3B had the highest sensitivity and specificity for predicting CCa development. Taken together, the current data showed that FNDC3B and BPGM were key genes involved in HPV-mediated transformation from normal epithelium to precancerous lesions and CCa.

## Introduction

Cervical carcinoma (CCa) is the fourth most commonly diagnosed and fatal gynecological cancer in the world (1). There were more than 600,000 new cases and 340,000 CCa-related deaths worldwide in 2020 (1). In China, there were 106,400 new cases in 2018, approximately 34% of which are ultimately fatal (2, 3). In the US, there were nearly 13,800 cases of invasive CCa and estimated 4,290 deaths of CCa in 2020 (4). Human papillomavirus (HPV) infection is the major cause of the development of CIN and CCa (5–7). Although HPV vaccine and early screening effectively reduce the incidence and mortality, the majority of CCa is diagnosed in advanced stages (8). The prognosis of advanced and metastatic CCa patients is poor with a mean survival rate of only 8 to 13 months (9). Therefore, it is indispensable to identify novel biomarkers to predict the occurrence of CIN and CCa.

Cervical carcinogenesis is a multistep process triggered by HPV infection (most prominently HPV16 and HPV18) (10). Viral oncoproteins (E6, E7, etc.) exert a crucial role in inducing the transformation of normal cells to malignant cells. The effect of E6/E7 oncoproteins on triggering malignant conversion is multifaceted, including inducing genomic instability, regulating tumor-associated gene expression, and accelerating cell cycle progression (11). E6-induced degradation of tumor-suppressor protein p53 destroys its biological roles in controlling cell cycle progression and facilitating programmed cell death, thus resulting in accelerated tumor cell growth (11). E6 oncoprotein also activates telomerase in epithelial cells through upregulating the expression of human telomerase reverse transcriptase (hTERT) (12). E7-induced loss of retinoblastoma protein (RB1) accelerates cell cycle progression by facilitating DNA synthesis (13). E6 and E7 also trigger chromosomal aberrations *via* inducing aberrant centrosome duplication (12).

Generally, HPV-associated cervical carcinogenesis is separated into four phases: (i) the prolonged life cycle due to E6/E7-triggered inhibition of p53 and RB1 (10, 12), (ii) the immortal phenotype due to E6/E7-induced chromosomal instability and telomerase activation (10), (iii) the anchorage-independent growth due to HPV-induced genetic and epigenetic changes (14), and (iv) tumorigenesis (complete transformation). The persistent HPV infection results in increasingly serious grades of dysplasia (CIN1, CIN2, CIN3) and eventually to CCa. Although this multistep process has been generally accepted, the molecular alterations behind these transitions remain poorly understood.

In the study, the GSE138080 data set was downloaded from the Gene Expression Omnibus (GEO) database. The mRNA expression profiles including normal, CIN, and CCa were obtained *via* analyzing the dataset. Based on the differential expression analysis and WGCNA analysis of an integrated dynamic process from normal to CIN, and CCa, we found that FNDC3B and BPGM could differentiate all stages in a stepwise progression of CCa. A higher expression of FNDC3B or lower expression of BPGM was closely associated with a shorter OS and DFS.

## Materials and Methods

### Data Acquisition

To identify the molecular alternations in the stepwise progression of HPV-associated CCa, the GEO database (https://www.ncbi.nlm.nih.gov/gds/?term=gse) was used to acquire mRNA expression profiles in cervical samples spanning from normal, CIN, and CCa. The GSE138080 dataset which included 8 healthy cervical, 12 CIN2/3, and 10 CCa tissues was used as the training dataset. The GSE63514 dataset included 24 normal, 14 CIN1, 22 CIN2, 40 CIN3, and 28 CCa tissues and was used as the validation dataset. A series of matrix files were downloaded from the GEO database. After preprocessing with background correction, data normalization, and principal component analysis (PCA) (**Supporting Figure S1**), a total of 12,524 genes were obtained from the GSE138080 to construct subsequent analysis.

The mRNA expression profiles and clinical information of 292 CCa samples were downloaded from the Cancer Genome Atlas (TCGA) database (https://cancergenome.nih.gov/). The association of FNDC3B or BPGM level with overall survival (OS) and disease-free survival (DFS) in patients with CCa was analyzed using the TCGA data.

### Construction of a Co-Expression Network

A total of 12,524 genes were used to construct a gene co-expression network using the WGCNA package in R software (15, 16). To ensure a scale-free characteristic, the appropriate soft threshold power parameter (β value) was first calculated through the function of pickSoftThreshold. Here, the soft threshold power β value was set as 12, in which the scale R^2^ > 0.8 and mean connectivity < 100. Secondly, Pearson’s association coefficient matrix was used to estimate the similarity of mRNA expression profiles, and then the expression matrix was converted to an adjacency matrix using the WGCNA R package. After the adjacency matrix was converted to topological overlap matrix (TOM), the hierarchical clustering was carried out to classify similar genes into the same module in compliance with the TOM-based dissimilarity measured with a minimum size of 40 for the gene dendrogram.

### Module-Trait Relationship Analysis and Hub Gene Identification

The relationships between modules and clinical traits were calculated in accordance with the phenotypic information of normal, CIN, and CCa groups. Two parameters, gene significance (GS) and module membership (MM), were calculated to quantify all genes on the array to each module. GS indicated the association of gene expression with clinical trait. MM indicated the association of gene expression with module eigengene. Based on the cutoff criteria (absolute value of MM > 0.8 and absolute value of GS > 0.2), genes with high connectivity in each module were identified as hub genes.

### Venn Diagram Analysis

Based on the cutoff criteria of |FC| > 1.5 and p < 0.05, differentially expressed genes (DEGs) were screened out between any two groups (normal vs. CIN, CIN vs. cancer, and normal vs. cancer). Then, DEGs between any two groups were delivered to the Venn diagram (http://bioinformatics.psb.ugent.be/webtools/Venn/) to obtain co-upregulated or co-downregulated genes, as previously described (17).

### Functional Enrichment Analyses

To reveal the biological processes of the genes in each module, gene ontology (GO) functional enrichment analysis was conducted through DAVID v6.8 (the Database for Annotation, Visualization, and Integrated Discovery), an online bioinformatics tool (http://david.abcc.ncifcrf.gov/) (18). GO categories with a false discovery rate < 0.05 were deemed as significant. To identify the pathways of the genes in each module, Kyoto Encyclopedia of Genes and Genomes (KEGG) pathway enrichment analysis was carried out using DAVID v6.8, and KEGG pathways with a false discovery rate < 0.05 were deemed as significant.

### Expression Analysis of FNDC3B and BPGM in Cervical Samples Spanning From Normal, CIN, to CCa

A GSE138080 dataset, which includes 10 normal controls, 15 CIN2/3, and 10 CCa tissues, was used as the training dataset to analyze the expression of FNDC3B and BPGM in stepwise progression of CCa. A GSE63514 dataset, which includes 24 normal controls, 14 CIN1, 22 CIN2, 40 CIN3, and 28 CCa tissues, was used as the validating dataset to analyze the expression of FNDC3B and BPGM. Forty-eight cervical samples, which include 14 normal, 15 CIN1-3, and 19 CCa, were collected from the Seventh Affiliated Hospital affiliated to Sun Yat-sen University with informed consents. These samples were obtained with the approval of the Hospital’s Protection of Human Subjects Committee. The expression of FNDC3B and BPGM in these tissues was assessed using quantitative real-time PCR (qRT-PCR).

### qRT-PCR

Forty-eight cervical samples (14 normal, 15 CIN1-3, and 19 CCa) were obtained from the Seventh Affiliated Hospital affiliated to Sun Yat-sen University with the approval of the Hospital’s Protection of Human Subjects Committee. Informed consents were obtained from these donors. Total RNAs were isolated from cervical tissues with TRIzol reagent (Invitrogen, Carlsbad, CA). The cDNA was synthesized from 1 µg total RNA using M-MLV reverse transcriptase (Invitrogen) and oligo(dT)18 primers in accordance with the manufacturer’s protocol. qRT-PCR was carried out in triplicate with SuperScript™ III Platinum™ SYBR™ Green (Invitrogen) on a 7500 Real-Time PCR System (Thermo Fisher Scientific, MA, USA). The temperature protocol was 95°C for 20 min, followed by 40 cycles (95°C for 15 s, 59°C for 15 s). Beta-actin was used as an internal control. The relative expression of FNDC3B and BPGM was calculated using the 2^-ΔΔCT^ formula (19). The sequence-specific primer pair for FNDC3B was 5′-CAACAGCCCTCCTTCTTCTATCT-3′ (sense) and 5′-GCACCCTCTTTACTTCCAACTCAT-3′ (antisense). The sequence-specific primer pairs for BPGM was 5′-ATCAGAAACTCAACAGCGAAGG-3′ (sense) and 5′-TGTGAATGGACCGATTAAGGAC-3′ (antisense). The sequence-specific primer pair for beta-actin was 5′-CCACGAAACTACCTTCAACTCC-3′ (sense) and 5′-TCTTGATCTTCATTGTGCTGGG-3′ (antisense).

### Mutation Analysis

Mutation analysis (158 samples in TCGA database) was carried out through the cBio Cancer Genomics Portal (http://cbioportal.org) (20, 21).

### Survival Analysis

Based on the median value of FNDC3B expression, CCa patients in the TCGA database were separated into high- and low-FNDC3B expression groups. OS analysis was carried out using the GEPIA online tool (http://gepia.cancer-pku.cn/index.html) to calculate the association of FNDC3B expression with survival time. DFS analysis was carried out to calculate the association of FNDC3B expression with disease-free survival time. Similarly, OS and DFS analysis was carried out to calculate the association of BPGM expression with survival time and disease-free survival time.

### Receiver Operating Characteristic Analysis

To evaluate the accuracy and specificity of FNDC3B and BPGM, receiver operating characteristic (ROC) and area under the curve (AUC) analyses were carried out using the GSE138080 dataset.

### Statistical Analysis

Data are represented as mean ± standard deviation (SD) of three independent replications. Statistical analysis was carried out with GraphPad Prism (GraphPad Software, Inc., CA, USA) and R software. The differences were deemed as significant when *p* was less than 0.05.

## Results

### Construction of WGCNA

**Figure 1:**
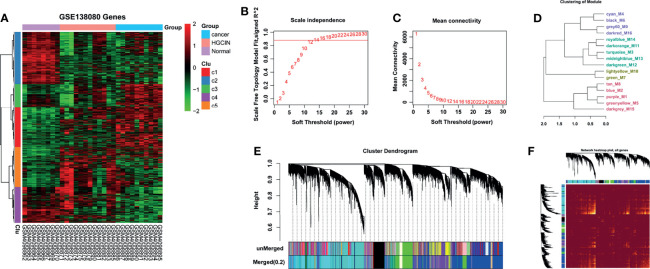
WGCNA on the dynamic progression of HPV-associated CCa. **(A)** Hierarchical clustering analysis of mRNAs differentially expressed in cervical samples spanning from normal, to CIN and CCa (fold change > 1.5 and *p* < 0.05). Eight normal, 12 CIN, and 10 CCa tissues in GSE138080 were used to conduct bioinformatics analysis after PCA. **(B, C)** Network topology analysis at different soft-thresholding powers. R2 > 0.8 and mean connectivity < 100 when β = 12, indicating that the co-expression network was a scale-free topology. **(D)** Cluster dendrogram based on module eigengenes. **(E)** The cluster dendrogram of co-expression network modules. The mRNAs with highly similar expression patterns were placed in the same module using the adaptive branch pruning algorithm. **(F)** An adjacency heatmap showed the topological overlap matrix among all 12,524 genes.

To investigate the molecular alternations in stepwise progression of HPV-associated CCa, the GSE138080 dataset was downloaded from the GEO database to analyze the mRNA expression profiles in cervical samples spanning from normal, CIN, to CCa. A total of 12,524 genes were obtained and used to construct a gene co-expression network with the WGCNA R software package (**Figure 1A** and **Supporting Table S1**). It is crucial to determine the soft-thresholding power in WGCNA to increase the co-expression similarity to figure out the adjacency (22). As shown in **Figures 1B, C**, the scale R^2^ was more than 0.8 and the mean connectivity was less than 100 when soft threshold power β = 12, suggesting that the co-expression network exhibited scale-free characteristics. The mRNAs with a highly similar expression pattern were placed in the same module using the adaptive branch pruning algorithm, and 16 modules were generated (**Figures 1D, E** **)**, ranging in size from 41 to 3,444 genes (**Table 1**). An adjacency heat map showed the topological overlap matrix among all of 12,524 genes, suggesting that every module exhibited independent validation to each other (**Figure 1F**).

**Table 1:** The number of mRNAs in the 16 modules.

No.	Module	Genes	Correlation, p value (MM vs. GS)
**1**	blue_M2	3444	0.061, 0.00034
**2**	turquoise_M3	2144	0.52, 7.7e-149
**3**	cyan_M4	1722	-0.047, 0.048
**4**	green_M7	1321	0.62, 3.9e-141
**5**	purple_M1	1262	0.78, 1e-200
**6**	black_M6	636	0.26, 2.8e-11
**7**	greenyellow_M5	451	0.76, 4.7e-86
**8**	midnightblue_M13	312	0.63, 6.5e-3
**9**	tan_M8	290	0.75, 1.2e-53
**10**	grey60_M9	230	0.83, 9.8e-60
**11**	lightyellow_M10	194	0.8, 1.8e-44
**12**	royalblue_M14	180	0.072, 0.34
**13**	darkred_M16	99	0.72, 4.5e-17
**14**	darkgreen_M12	95	0.57, 1.6e-09
**15**	darkgrey_M15	53	0.055, 0.07
**16**	darkorange_M11	41	-0.45, 0.0032

**Figure 2:**
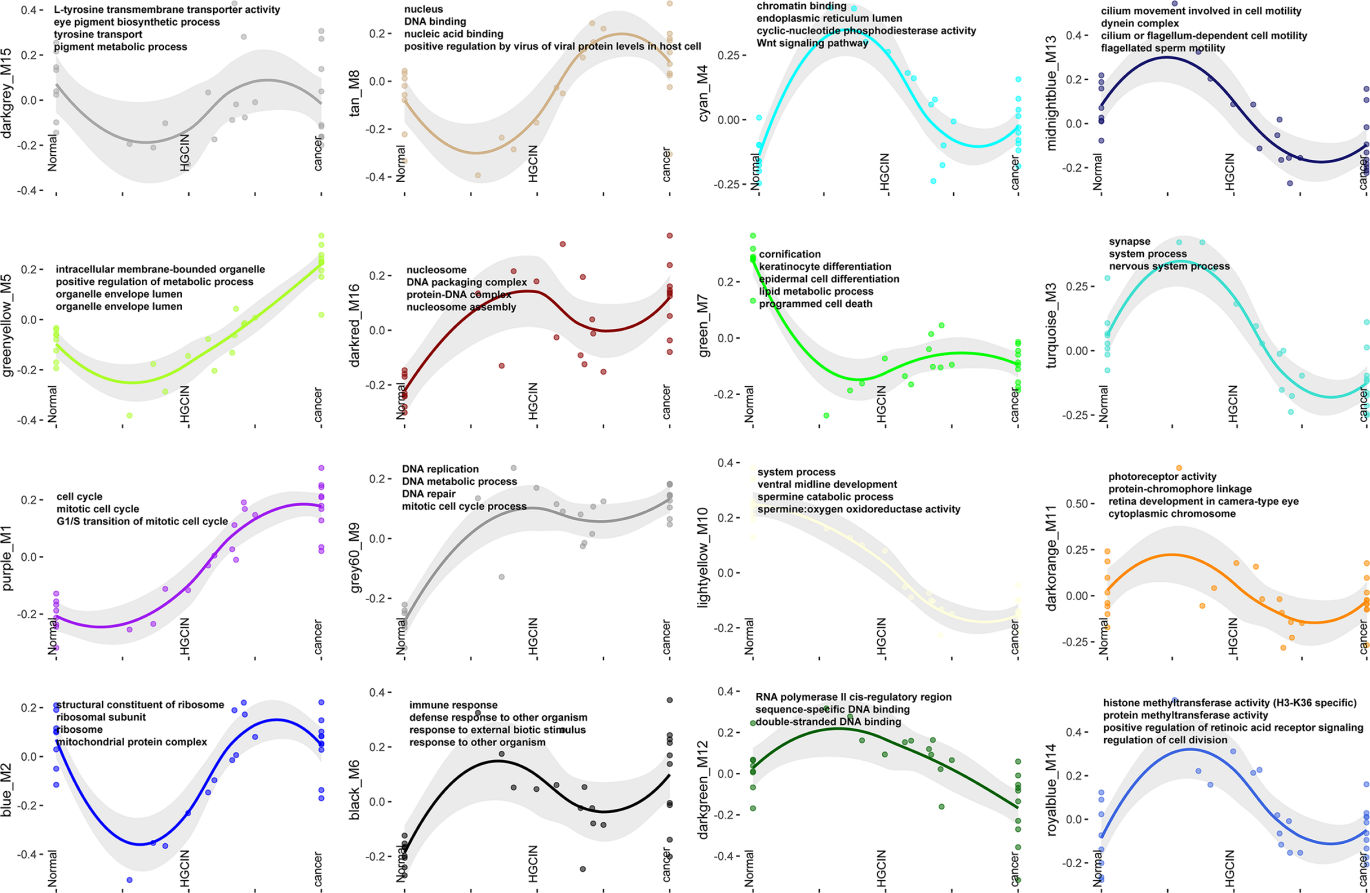
GO functional enrichment analysis. GO terms associated with each of the 16 modules.

GO analysis exhibited several critical biological processes involved in carcinogenesis (**Figure 2**). The GO terms of the purple_M1 module showed that several genes involved in the cell cycle (*AHR*, *XIAP*, *BAX*, *BCL6*, *CASP2 CDK1*, and *CCNE1*, etc.), DNA replication (*ATR*, *BARD1*, *BLM*, *BRCA1*, *BRCA2*, *CCNA2*, and *CCNE1*, etc.), and telomere maintenance (*PCNA*, *POLE2*, *PRIM1*, *PRIM2*, and *RFC2*, etc.) were persistently increased after HPV infection. The GO terms of the green_M7 module exhibited that several genes involved in cornification (*CSTA*, *DSC2*, *DSG1*, *DSP*, *FLG*, and *KRT1*, etc.) and lipid metabolic process (*ACAA1*, *ACADSB*, *ACAT1*, *ACOX1*, and *AKR1B1*, etc.) were decreased after HPV infection and remained at a low level in CCa. GO terms of the grey60_M9 module showed that several genes involved in DNA replication (*CDC6*, *DDX11*, *DUT*, *LIG1*, *MCM3*, *MCM5*, and *MCM7*, etc.) and DNA metabolic process (*PARP1*, *FANCA*, *FANCC*, *FANCD2*, *HELLS*, *KIF22*, *TCF7*, and *TK1*, etc.) were persistently increased after HPV infection. The significance of other biological processes remains to be clarified, although dysregulated cell cycle, DNA replication, and telomere maintenance were correlated with carcinogenesis (23–25).

### Relationship Between Key Modules and Different Stages in CCa

**Figure 3:**
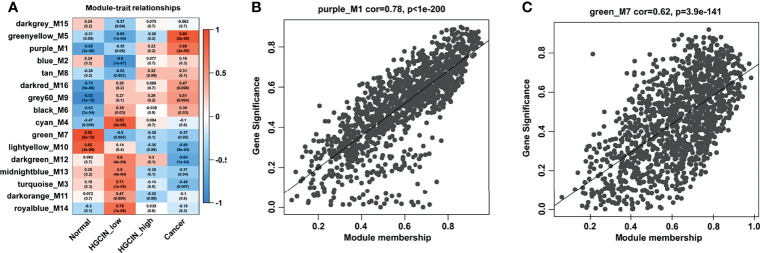
The relationship between modules and clinical traits. **(A)** Module-trait relationship analysis. Each row indicated a module eigengene, and each column indicated a clinical trait. Each cell included the corresponding correlation and p value. **(B)** A scatterplot of gene significance (GS) vs. module membership (MM) in the purple_M1 module (correlation = 0.78, p < 1e-200). **(C)** A scatterplot of GS vs. MM in the green_M7 module (correlation = 0.62, p < 3.9e-141).

The association of each module with clinical traits was analyzed. The r and p values were set in the heat map of the module–trait relationship. Based on module size (**Table 1**), the correlation between gene significance (GS) and module membership (MM) (**Table 1**), and r and p values (**Figure 3A**), purple module (M1) (r = -0.68, p = 3e-05) and green module (M7) (r = 0.92, p = 5e-13) were identified as two key modules closely related to HPV-associated CCa. After filtering through GS > 0.2 and MM > 0.8, 191 genes in the M1 and 215 genes in the M7 were identified as hub genes, respectively (**Supporting Tables S2** and **3** **).** The scatterplot between GS and MM in the M1 showed that the GS value was markedly associated with the MM value (cor = 0.78, p = 1e-200, **Figure 3B**). The scatterplot between GS and MM in the M7 showed that the GS value was markedly associated with the MM value (cor = 0.62, p = 3.9e-141, **Figure 3C**). These data suggested that these hub genes in the M1 and M7 modules were highly correlated with disease progression.

### Functional Annotation of M1 and M7 Modules

**Figure 4:**
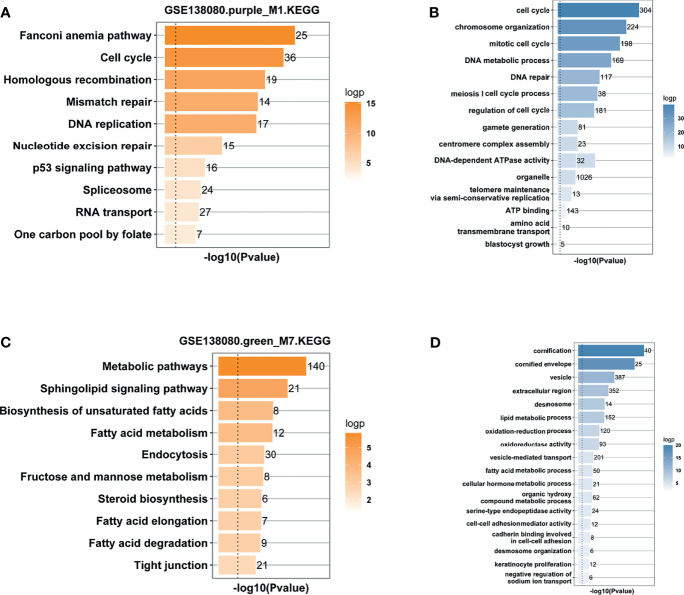
Functional enrichment analysis. **(A)** KEGG enrichment analysis of genes in the purple_M1 module. **(B)** GO enrichment analysis of genes in the purple_M1 module. **(C)** KEGG enrichment analysis of genes in the green_M7 module. **(D)** GO enrichment analysis of genes in the green_M7 module.

Genes in the M1 and M7 modules were used to conduct GO and KEGG pathway enrichment analysis, respectively. The results from KEGG analysis showed that DNA damage response (DDR, including Fanconi anemia, and mismatch repair pathways), cell cycle, p53 signaling pathway, and metabolic pathway (sphingolipid signaling pathway and fatty acid metabolism) were enriched (**Figures 4A, C** **)**. In fact, the E6-induced degradation of the p53 protein accelerates cell cycle progression and represses programmed cell death (11). The Fanconi anemia pathway is a portion of DDR, and HPV infection increases the activation of the Fanconi anemia pathway (26). The results from GO analysis showed that genes in the two modules were largely correlated with the biological processes of the cell cycle, DNA repair, telomere maintenance, and fatty acid and lipid metabolic process (**Figures 4B, D** **)**. Interestingly, 191 hub genes in the M1 module and 215 hub genes in the M7 were used to carry out KEGG analysis, and results showed that hub genes in the two modules were also mainly enriched in cell cycle, Fanconi anemia and mismatch repair pathways, p53 signaling pathway, and metabolic pathways (**Supporting Figure S2**).

### FNDC3B and BPGM Distinguished Different Stages of Progression of CCa

**Figure 5:**
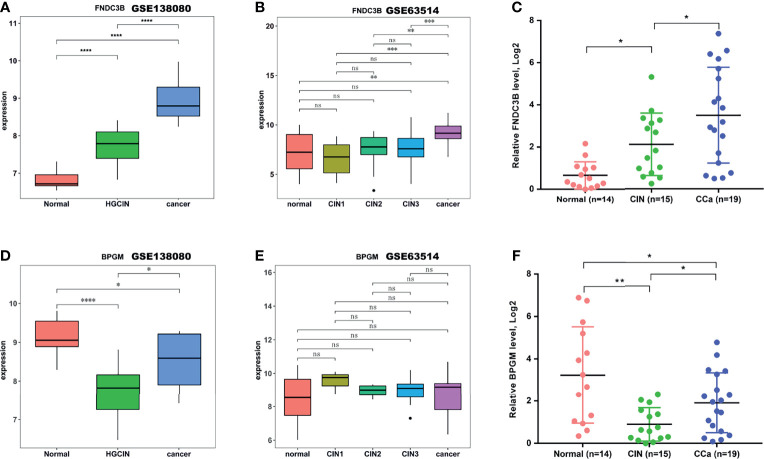
FNDC3B and BPGM expression analysis. **(A, B)** The expression of FNDC3B analyzed in the test dataset GSE138080 and in the validation dataset GSE63514. **(C)** The expression of FNDC3B assessed using qRT-PCR in clinical specimens. **(D, E)** The expression of BPGM analyzed in the test dataset GSE138080 and in the validation dataset GSE63514. **(F)** The expression of BPGM assessed using qRT-PCR in clinical specimens. *p < 0.05, **p < 0.01, ***p < 0.001, ****p < 0.001, ns, not significant.

To identify the key genes in the M1 and M7 modules that can distinguish different stages in the stepwise progression of CCa, a Venn analysis was first carried out to screen out co-dysregulated genes between any two groups (CIN vs. normal, cancer vs. normal, and cancer vs. CIN). There were 111 co-upregulated genes and 78 co-downregulated genes (**Supporting Figures S3A, B**). According to these co-dysregulated genes and hub genes in the M1 (or M7) module, 63 overlapping genes were identified as key genes in the M1 module and 79 overlapping genes were identified as key genes in the M7 module (**Supporting Table S4**). FNDC3B in the M1 module was increasingly increased in the stepwise progression of CCa (**Figure 5A**). In another dataset (GSE63514), the FNDC3B expression can differentiate different stages in the stepwise progression of CCa (**Figure 5B**). To further validate these results, 48 cervical samples (14 normal, 15 CIN1-3, and 19 CCa) were collected to assess FNDC3B expression. **Figure 5C** showed that the expression of FNDC3B was remarkably increased in the stepwise progression of CCa, from normalcy to CIN and CCa. In addition, BPGM in the M7 module could differentiate all stages of progression of CCa in GSE138080 (**Figure 5D**), although the BPGM did not change between the three groups (**Figure 5E**). In 48 cervical samples, BPGM could differentiate all stages in the stepwise progression of CCa (**Figure 5F**).

### Mutation Analysis of *FNDC3B* and *BPGM* Genes

**Figure 6:**
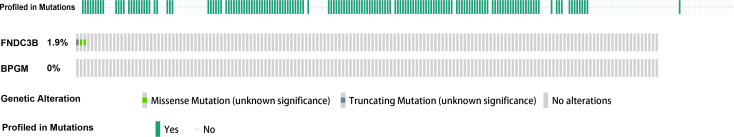
Mutation analysis of FNDC3B and BPGM in CCa. There were 1.9% genomic alterations of FNDC3B in CCa, and there were 0% genomic alterations of BPGM in CCa.

Given that the expression of FNDC3B and BPGM was dysregulated in CCa, we next assessed the association of the aberrant expression of FNDC3B and BPGM with genomic change. No mutation of *FNDC3B* and *BPGM* genes was found in most patients by analyzing the TCGA database with the cBioPortal tool. These data suggest that the aberrant expressions of FNDC3B and BPGM exert a critical role in HPV-associated cervical carcinogenesis (**Figure 6**).

### Aberrant Expressions of FNDC3B and BPGM Were Correlated with OS and DFS in CCa Patients

**Figure 7:**
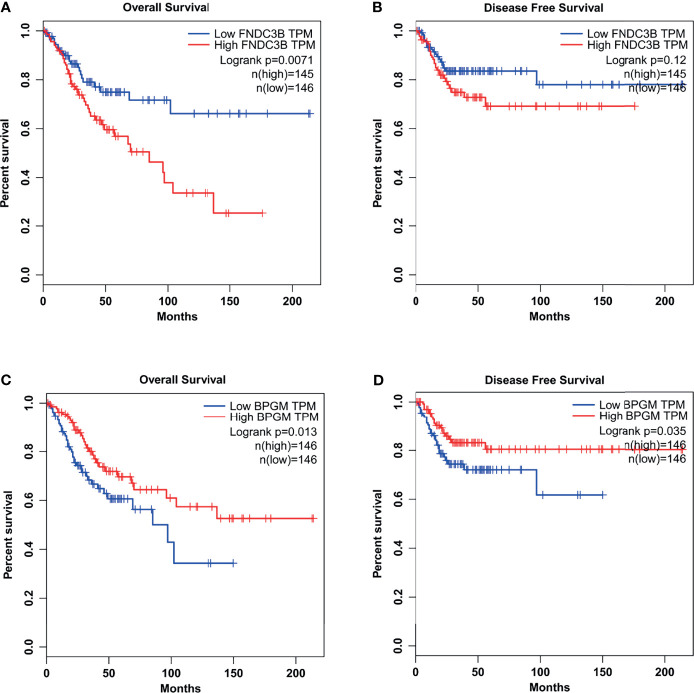
The relationships between FNDC3B (or BPGM) expression and the clinical outcome in CCa patients. **(A)** A higher expression of FNDC3B exhibited a significantly worse OS. **(B)** A higher expression of FNDC3B exhibited a worse DFS. **(C, D)** A lower expression of BPGM exhibited a significantly worse OS and DFS.

Interestingly, the expression of FNDC3B was closely correlated with the clinical outcome in CCa patients. Through TCGA database analysis, we found that a higher expression of FNDC3B exhibited a significantly worse OS (**Figure 7A**). Disease-free survival (DFS) was also shorter in patients with high FNDC3B expression, compared with low FNDC3B expression (**Figure 7B**). The expression of BPGM was correlated with the clinical outcome in CCa patients. As shown in **Figures 7C, D**, a lower expression of BPGM exhibited a significantly worse OS and DFS.

### FNDC3B Had the Highest Sensitivity and Specificity for Predicting CCa

**Figure 8:**
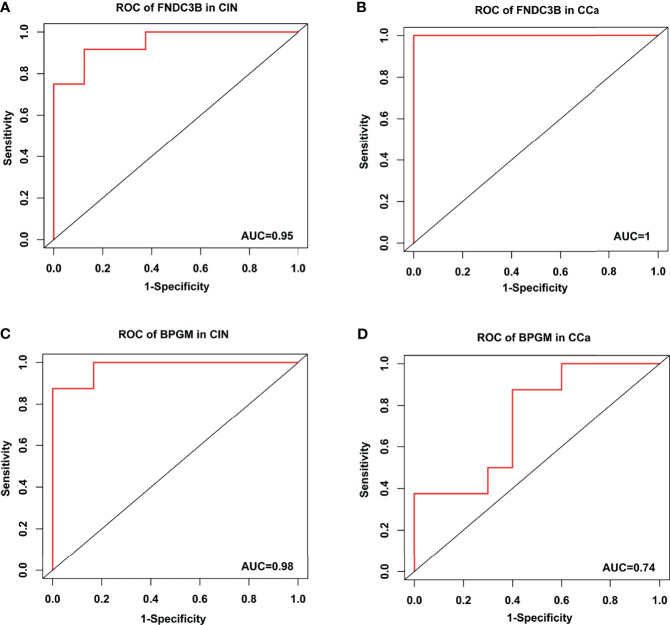
Receiver operating characteristic curve analysis in CIN and CCa. **(A)** ROC of FNDC3B in CIN. **(B)** ROC of FNDC3B in CCa. **(C)** ROC of BPGM in CIN. **(D)** ROC of BPGM in CCa.

To assess the accuracy and specificity of FNDC3B, an ROC analysis of CIN and CCa was carried out using GSE130808. The AUCs of CIN and CCa were 0.95 and 1.0, respectively (**Figures 8A, B** **)**. The accuracy and specificity of BPGM were also assessed using ROC analysis. The AUCs of CIN and CCa were 0.98 and 0.74, respectively (**Figures 8C, D** **)**. These results demonstrate that FNDC3B and BPGM be used as prognostic biomarkers for the early diagnosis and prognosis of CCa.

## Discussion

HPV-induced malignant transformation from normal cervical epithelial cells to cancer cells is a multistep and progressive process (27, 28), in which viral oncoprotein-driven molecular alterations are essential to carcinogenesis (10). It was known that E6 oncoprotein binds to E3 ubiquitin ligase E6AP (E6-associated protein) to induce p53 ubiquitination and results in a subsequent proteasomal degradation of p53 (29). As a result, p53 inactivation leads to a decreased cell apoptosis and accelerates cell survival and proliferation. E7 disrupts the Rb/p16 pathway and thus allows cells to evade G1/S checkpoint arrest (30). E7 can also enhance E6-induced telomerase activation by increasing hTERT expression (31). At present, a large number of dysregulated genes are identified in HPV-CCa (32–34). However, most studies focused on the differentially expressed genes between normal and CCa tissues, and molecular alterations spanning from HPV infection, to CIN and CCa, have rarely been investigated.

With the advancement of microarray, next-generation sequencing techniques, and bioinformatics algorithms, great progresses have been made in systemically analyzing the molecular alterations in the stepwise progression of CCa. Den Boon et al. analyzed the mRNA expression profile in cervical tissues including normal, CIN1, CIN2, CIN3, and CCa and identified a series of progressive alterations (28). Genes involved in DNA replication/repair and cell cycle are upregulated in CIN1/2 compared with normal samples and maintained at CIN3 and CCa. Genes involved in metabolic processing, protein synthesis, and inflammatory response are upregulated in normal-to-CIN transition and then turned down in CIN-to-CCa transition (28). Estrogen receptor (ER) α is markedly and progressively downregulated in the stepwise progression of CCa (28). Given the crucial role of ERα in the occurrence and progression of CCa (35, 36), this study demonstrates that ERα could act as a reliable biomarker for the early diagnosis and prognosis of CCa.

In this study, a powerful system biology algorithm, WGCNA, was used to analyze the associations among genes, and then similar genes were classified into modules and these modules were correlated with phenotypic traits (22, 37). At present, WGCNA has been applied to identify diagnostic biomarkers and therapeutic targets in different types of tumors (21, 38, 39). Through analyzing the GSE138080 dataset, 16 modules were generated. Based on module size, the correlation between modules and phenotypic traits, and the correlation between GS and MM, the M1 and M7 modules were identified as two key modules closely related to CCa. Genes in the M1 module were remarkably increased in the stepwise progression of CCa. These genes are involved in cell cycle, nuclear division, DNA replication/repair, and cell division. FNDC3B in the M1 module exhibits a marked and progressive increase in the stepwise progression of CCa. More importantly, a higher expression of FNDC3B results in a worse OS and DFS. FNDC3B is an oncogene firstly identified in hepatocellular carcinoma (HCC) (40). Forced expression of FNDC3B promotes malignant transformation of normal hepatocytes and mammary and kidney epithelial cells through activating Rb1, transforming growth factor-β, and phosphoinositide 3-kinase/Akt signaling (41). In cervical squamous cell cancer, FNDC3B inhibition hampers cancer cell proliferation and invasion (42). Genes in the M7 module were decreased after HPV infection and remained at a low level in CCa. These genes are correlated with epidermal cell differentiation, metabolic pathway, and sphingolipid pathway. BPGM in M7 could distinguish all stages in the stepwise progression of CCa. Moreover, a lower expression of BPGM results in a significantly worse OS and DFS. However, the BPGM expression is increased HCC, and upregulated BPGM is associated with a poor prognosis in HCC patients (43). Further studies are needed to validate the role of FNDC3B and BPGM as biomarkers. Additionally, the biological function of FNDC3B and BPGM in regulating cancer cell proliferation, invasion, and therapy resistance needs to be clarified.

## Conclusion

This study suggests that FNDC3B and BPGM could be reliable biomarkers for the early diagnosis and prognosis of CCa. The results of the study will help the management of HIV-induced carcinogenesis of CCa.

## Data Availability Statement

The original contributions presented in the study are included in the article/**Supplementary Material**. Further inquiries can be directed to the corresponding authors.

## Author Contributions

YX and QW contributed toward the concept, design, and statistical analysis of the research work. LZ and HY conducted the literature search, carried out the experimental studies, and prepared the manuscript. TD, LL, and RO reviewed and edited the manuscript. ML and JW helped with the experimental part. All authors contributed to the article and approved the submitted version.

## Funding

This work was supported by the National Natural Science Foundation of China (Nos. 81771531, 81871129, 82072862, 82072863, and 82002727).

## Conflict of Interest

The authors declare that the research was conducted in the absence of any commercial or financial relationships that could be construed as a potential conflict of interest.

## Publisher’s Note

All claims expressed in this article are solely those of the authors and do not necessarily represent those of their affiliated organizations, or those of the publisher, the editors and the reviewers. Any product that may be evaluated in this article, or claim that may be made by its manufacturer, is not guaranteed or endorsed by the publisher.
