# Supplementary figures and images for: FNDC3B and BPGM Are Involved in Human Papillomavirus-Mediated Carcinogenesis of Cervical Cancer
Source: Front Oncol. 2021 Dec 16;11:783868. doi: 10.3389/fonc.2021.783868 (PMC8716600; doi:10.3389/fonc.2021.783868)

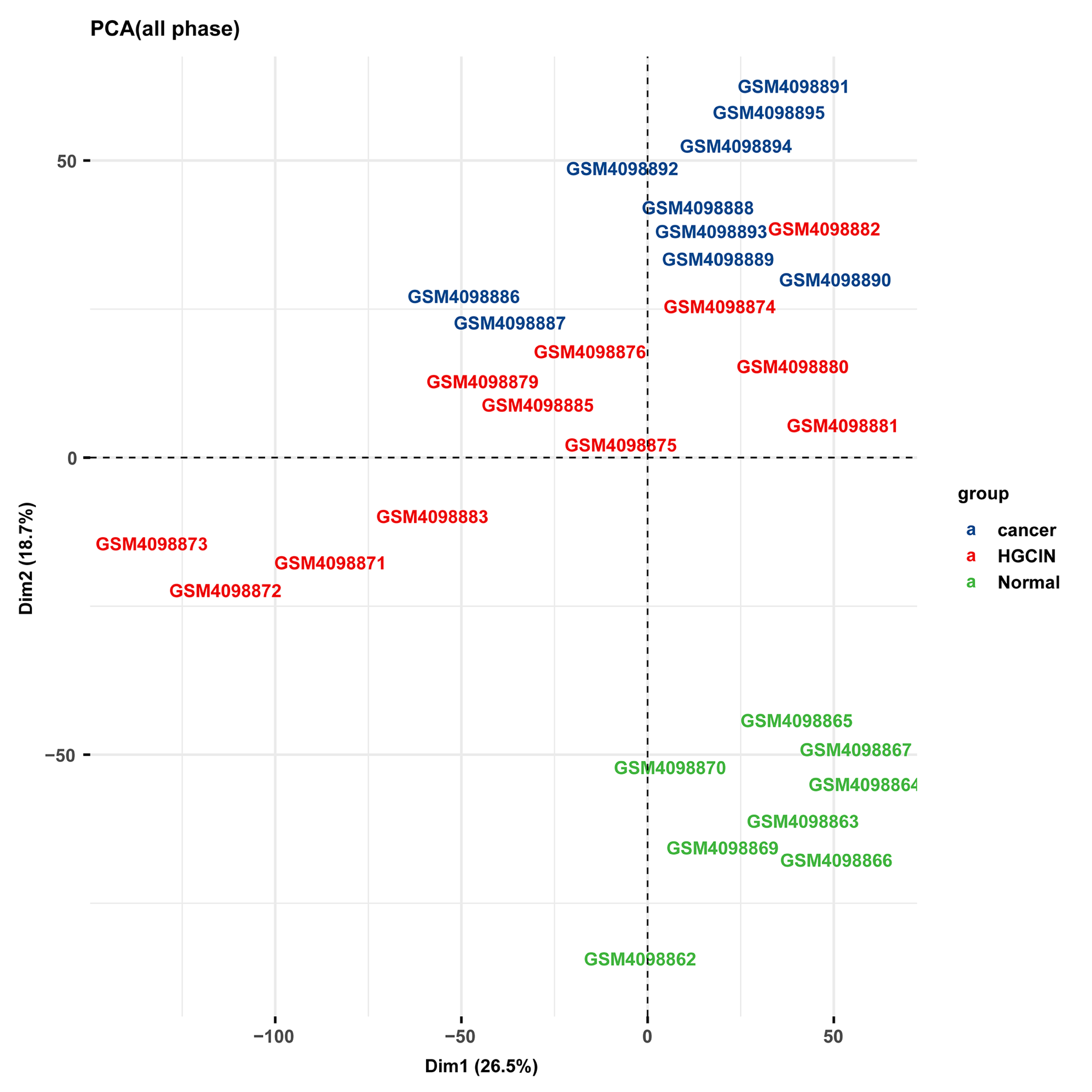

Supplement: Supplementary file 1 [file Presentation_1.zip › Supporting Figure S1.tif]

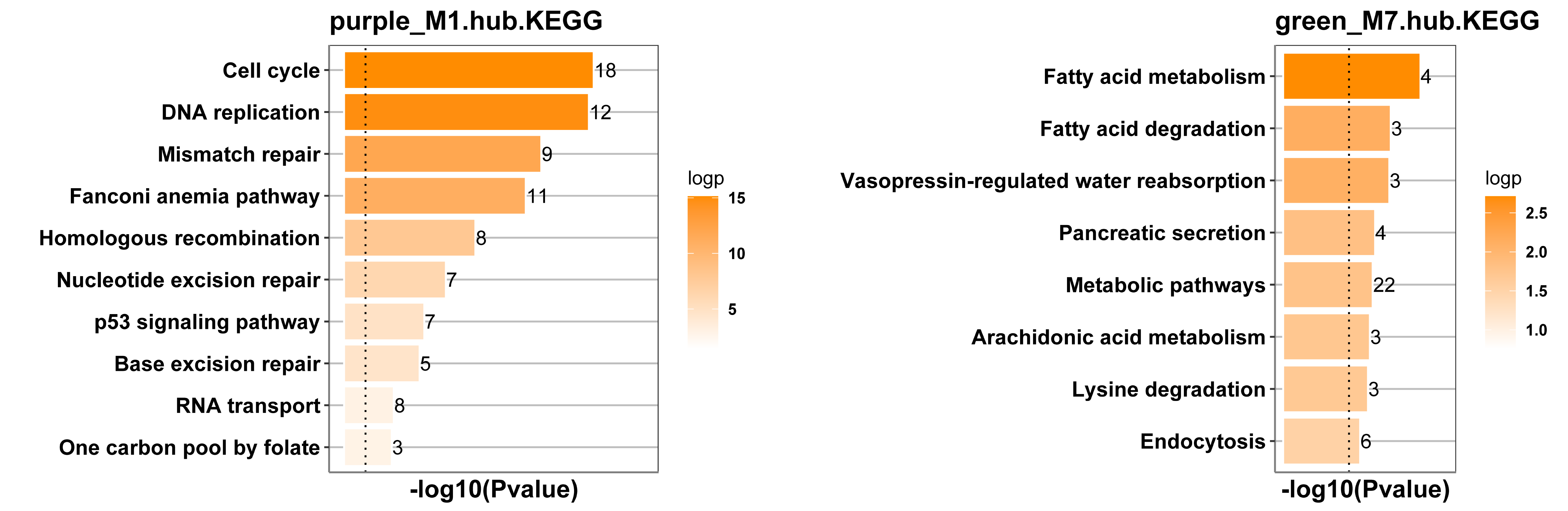

Supplement: Supplementary file 1 [file Presentation_1.zip › Supporting Figure S2.tif]

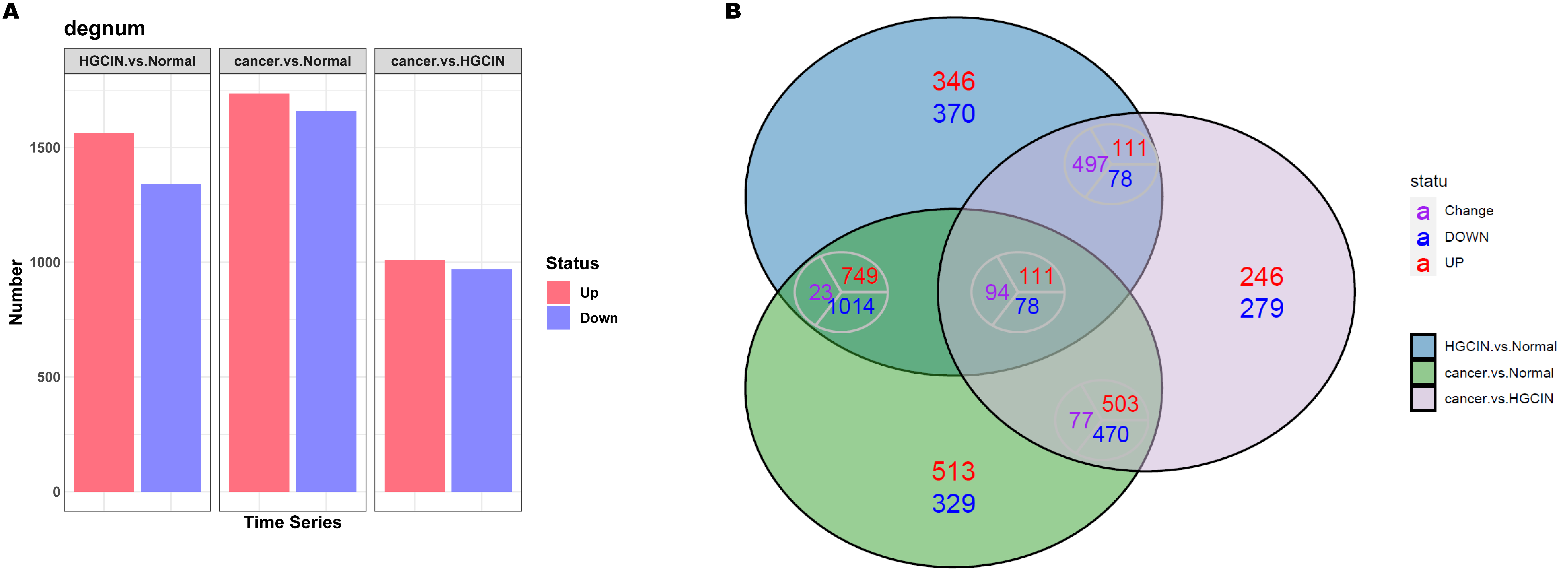

Supplement: Supplementary file 1 [file Presentation_1.zip › Supporting Figure S3.tif]
